# Inducible Expression of the *De-Novo* Designed Antimicrobial Peptide SP1-1 in Tomato Confers Resistance to *Xanthomonas campestris* pv. *vesicatoria*

**DOI:** 10.1371/journal.pone.0164097

**Published:** 2016-10-05

**Authors:** Areli Herrera Diaz, Izabella Kovacs, Christian Lindermayr

**Affiliations:** Institute of Biochemical Plant Pathology, Helmholtz Zentrum München–German Research Center for Environmental Health, 85764, München/Neuherberg, Germany; Bose Institute, INDIA

## Abstract

Antimicrobial peptides (AMPs) are small peptides with less than 50 amino acids and are part of the innate immune response in almost all organisms, including bacteria, vertebrates, invertebrates and plants. AMPs are active against a broad-spectrum of pathogens. The inducible expression of AMPs in plants is a promising approach to combat plant pathogens with minimal negative side effects, such as phytotoxicity or infertility. In this study, inducible expression of the *de-novo* designed AMP SP1-1 in Micro Tom tomato protected tomato fruits against bacterial spot disease caused by *Xanthomonas campestris* pv. *vesicatoria*. The peptide SP1-1 was targeted to the apoplast which is the primary infection site for plant pathogens, by fusing SP1-1 peptide to the signal peptide RsAFP1 of radish (*Raphanus sativus*). The pathogen inducibility of the expression was enabled by using an optimized inducible 4XW2/4XS promoter. As a result, the tomato fruits of independently generated SP1-1 transgenic lines were significantly more resistant to *X*. *campestris* pv. *vesicatoria* than WT tomato fruits. In transgenic lines, bacterial infection was reduced up to 65% in comparison to the infection of WT plants. Our study demonstrates that the combination of the 4XW2/4XS *cis*-element from parsley with the synthetic antimicrobial peptide SP1-1 is a good alternative to protect tomato fruits against infections with *X*. *campestris* pv. *vesicatoria*.

## Introduction

The production of antimicrobial peptides (AMPs) is a conserved mechanism of the innate immune system to protect organisms against pathogens. AMPs can be generated by many different species ranging from bacteria and plants over to mollusks and vertebrates. Generally, AMPs are small peptides, between 8 to 50 amino acids in size and can be categorized according to their amino-acid composition, size and conformation. The largest group of AMPs is the cationic peptides, which can be further subdivided into three groups. Alpha helical peptides like magainins and cecropins, stabilize β-sheet peptides with several cysteine residues to form disulphide bonds like defensins and peptides, which contain proline- and arginine rich regions [1,2].

The classical mode of action of AMPs is permeabilization of target cell membranes by interacting with negatively charged compounds, such as hydroxylated phospholipids or teichonic acids [3,4]. Recently, other mechanisms of action have been described, including inhibition of cell wall-, nucleic acids- and protein synthesis [5–8]. It could be shown that AMPs can have fungicide, bacteriostatic, bactericide, antiviral, antiparasitic and insecticidal activity with low or no cytotoxicity to mammalian cells [1,9–11].

During the past years new AMPs have been discovered and described and the Antimicrobial Peptide Database currently contains 2619 natural AMPs from six kingdoms (*http://aps.unmc.edu/AP*) [12]. Some of them can inhibit the growth of several different pathogens, including multidrug-resistant pathogens [13,14]. Resistance to AMPs appears to be less common than the resistance to conventional drugs. Nevertheless, some bacterial strains have developed mechanisms to neutralize natural AMPs [8]. Some bacteria have changed their bacterial cell surface, while others degrade and neutralize AMPs by proteases or proteins that specifically bind AMPs [15]. However, by varying their amino acid sequence, AMPs can be optimized resulting in enhanced resistance against degradation and increased antimicrobial activity [16]. It could be shown, that synthetic *de-novo* designed AMPs can protect animals and plants against infections [17,18]. To avoid the excess use of antibiotics and pesticides in agriculture, the generation of resistant plants is necessary. Natural or synthetic AMPs represent an option for the treatment or prevention of bacterial and fungal infections in plants. Previously we presented a set of more than 60 designed peptides, with serveral of them displaying antibacterial and antifungal against a broad range of phytopathogens, including *Pseudomonas corrugata*, *Cladosporium herbarum*, and *Xanthomonas campestris pv*. *vesicatoria*. The plant protecting activity was already demonstrated in tomato (*Solanum lycopersicum*) by spraying the AMPs directly on the leaves [19]. An alternative strategy would be the stable expression of AMPs in tomato plants using recombinant technologies. Nevertheless, it has been shown that constitutively expression of recombinant AMPs can be toxic for plants, causing several deleterious effects such as infertility, reduced growth or cell death [20]. Thus, expression of AMPs in an inducible way may be desired to avoid toxic effects. This could be already demonstrated by using heat shock inducible or pathogen inducible promoters [21,22].

Tomatoes are one of the most consumed and important crop worldwide (FAOSTAT; http://faostat.fao.org/). In general, tomato plant diseases can have several negative effects ranging from diminished overall crop yield to reduced storage time of the fruits. The tomato cultivar Micro Tom (MT) is known as a model system, which has been widely used for the understanding of plant development, physiology and pathology [23,24]. It has similar advantages as the model plant *Arabidopsis*: MT has a small size, with a short life cycle of two to three months. Regeneration and transformation methods have been established. In this study, this tomato cultivar was genetically modified expressing the *de-novo* designed AMP SP1-1 under control of a pathogen-inducible promoter. Enhanced resistance of these plants against *X*. *campestris* pv. *vesicatoria* could be demonstrated.

## Materials and Methods

Chemicals were purchased from Sigma Chemical Co., St Louis MO; PhytoTechnology Lab., Shawnee Mission, KS; USB Co., Cleveland Ohio; Roche Co., Mannheim; Invitrogen Co., Carlsbad, CA; Promega Co., Madison, WI; Stratagene, La Jolla, CA; Qiagen Inc, Valencia, CA; unless otherwise stated on the text. Micro Tom seeds were purchased from Kiepenkerl (Münster, Germany).

### Vector construction and *Agrobacterium*-mediated transformation

To direct the AMPs to the apoplastic space, the signal peptide sequence of the radish RsAFP1 defensine gene [25] was fused upstream to the coding sequences of an SP1-1 antimicrobial peptide. Codon selection was done according to tomato codon usages frequency (www.kasuza.or.jp/codon/). The nucleotide sequence containing the FF::RsAFP1-SP1-1::NOS cassette was chemically synthesized (GeneArt® Gene Synthesis from Invitrogen). This cassette contains a 35 S minimal promoter with a *Xho*I restriction site at the 3´end and two *cis*-acting element F (Heise et al 2002) flanked by *Sac*I, *Apa*I-*Xba*I at the 5´end [26]. The coding sequences for the signal peptide of RsAFP1 and synthetic peptide SP1-1 were flanked by attB1-attB2 elements to allow subsequent cloning with the Gateway system. Finally, at the 3´end the terminator of the nopaline synthase (NOS) gene was introduced using *EcoR*V and *Hind*III restriction sites downstream of the coding sequence.

The nucleotide sequence of the minimal 35S promoter and the 4XW2 (5`-TTATTCAGCCATCAAAAGTTGACCAATAAT-3`) *cis*-acting element from the PR1 parsley gene [27] and the 4X box S (5`-CAGCCACCAAAGAGGACCCAGAAT-3`) from the parsley EL17 genes [28] were also chemically synthesized (GeneArt® Gene Synthesis from Invitrogen). The synthetic plant promoters were designed as previously described [27]. *Sac*I-*Xba*I restriction sites were flanked by the *cis*-active elements and *Xba*I-*Xho*I the 35 S minimal promoter in the vector pMKRQ::4XW2/4XS.

The phosphomannose isomerase (PMI) gene from *Escherichia coli* was used as a selection marker, for those constructs. DNA from *E*. *coli* strain XL-1-Blue was extracted with a genomic DNA isolation kit from bacteria (Nexttec). The PMI coding sequence was amplified by PCR using an Accu Prime High fidelity Taq DNA polymerase (Invitrogen) following the manufacturer´s instructions. Two flanking XhoI sites were introduced in the primers 5´-GCActcgagCATGCAAAAACTCATTAACTCAG-3’ and 5’-GCActcgagC TCTTACAGCTTGTTGTAAAC-3’ as described elsewhere [29]. The hygromycin phosphotransferase (HygR) cassette of pCambia 1300-nLuc was cloned into a pGemT-easy vector (Promega) to facilitate subsequent cloning. The HygR gene was removed by *Xho*I and replaced with the *Xho*I flanked PMI gene. Positives and right oriented clones were characterized with restriction enzymes and positive clones were sequenced. A binary vector pPm42GW was used to replace the attR4-ccdB-attR2 region (*Sac*II-*EcoICR*I) with PMI cassette (*Sac*II-*BsrB*I). The new PMIGW vector was characterized with restriction enzymes. The FF::RsAFP1-SP1-1::NOS cassette was sublcloned into the PMIGW vector using *Apa*I-*EcoR*V and *Apa*I-*Zra*I restriction sites respectively. The resulting plasmid was designated pPMIGW-FF-SP1-1. The pPMIGW-4XW2/4XS-SP1-1 was generated by replacing the FF element with the 4XW2/4XS element from the vector pMKRQ::4XW2/4XS using *Apa*I-*Xba*I restriction enzymes. Positive clones were characterized by restriction enzyme analysis and sequencing.

Generated vectors were transformed via electroporation into *Agrobacterium tumefaciens* pGV3101 containing the helper plasmid pMP90 (Gent^R^). Positive clones were grown overnight in 2 ml Luria-Bertani (LB) medium containing 50 mg/l rifampicin, 25 mg/l gentamicin and 50 mg/l spectinomycin.

For transient expression, a pBT10::4W2-GFP-GUS was generated. GFP from pB2GW7::RsAFP-GFP-GUS was amplified with *Xho* I-*Nco*I GFP (5´-CCGCTCGAGTGGCCACCATGGCTAAGTTTGCTTCTATTATTGC-3`) and GUS (5´-CAGGTGTTCGGCGTGGTGTAGAG-3´) Primers. The GFP PCR product was digested and cloned in *Nco*I-*Mun*I restriction sites of pBT10::4XW2-GUS plasmid. Plasmids were characterized by restriction analysis and sequencing.

### Protoplast isolation and transient transformation

Protoplasts from tomato TH3 calli cultures were isolated as described previously [30] with some modifications. Up to 2 g of TH3 calli were incubated for 2 hours in a protoplast wash solution (Sigma D9692) containing 2% Cellulase R10 and 1% Macerozyme. Released protoplasts were washed twice and set to 400,000 cells in 200 μl MMg buffer (0.4 M mannitol, 15 mM MgCl2, 4 mM MES pH 5.7). Ten μg of DNA (pBT10::4XW2-GFP-GUS) and 220 μl PEG solution (40% PEG4000 in ddH2O, 0.2 M mannitol, 100 mM CaCl_2_) were added to the protoplasts for each transfection experiment. After 30 minutes incubation on ice, transfected protoplasts were resuspended in 800 μl W5 buffer (2mM MES (pH5.7), 154 mM NaCl, 125 mM CaCl_2_ and 5 mM KCl) and washed two times. Finally, protoplasts were resuspended in 1 ml W5 buffer and divided for induction experiments. Induction was performed with Pep 25 (400 ng) or *X*. *campestris* pv. *vesicatoria* (1x10^6^ cfu/ml) for 14 to 18 hours. Two independent experiments with two replicates were performed.

### Microscopy

Protoplasts were analyzed for GFP fluorescence by Olympus BX61 fluorescence microscope (Olympus, Hamburg, Germany). eGFP was detected with a GFP filter at an excitation wavelength of 480 nm and 520 nm emission wavelength. Fluorescence intensity was measured using IMAGEJ software (US National Institutes of Health, Bethesda, MD, USA, http://imagej.nih.gov/ij/). The determined fluorescence densities were corrected by area and the background signal was subtracted from the corrected densities. Results are expressed in the percentage of GFP expressing protoplasts and the percentage of relative fluorescence intensity.

### Tomato regeneration and *Agrobacterium*-mediated trans-formation

Seeds of Tomato Micro-tom were surface-sterilized in 70% ethanol for 1 min and in 3% NaOCl with 20 μl Tween 20 for 20 min, and then rinsed five times 10 min each with sterile water. The seed were germinated on MS tissue culture medium (Murashige and Skoog 1962) containing 30 g/l sucrose and 8 g/l agar. The pH was adjusted to 5.8 ± 1 adjusted with either HCl or KOH and autoclaved at 121°C for 20 min. For the regeneration experiments, cotyledons from 7 days old seedlings were cut off in sterile conditions, the tips were removed and sectioned transversely in two fragments, and explants were incubated on three different callus induction media called as Preculture Media 1 to 3 for differentiation of tissues. Preculture Medium 1 (PM1) contained MS salts and vitamins, 30 g/l sucrose, 8 g/l agar, 1 mg/l naphthalene acetic acid (NAA) and 1 mg/l 6-benzylaminopurine (BA), pH 5.7±1 [31]. Preculture Medium 2 (PM2) contained MS salts and B5 vitamins 30 g/l sucrose, 8 g/l agar, 0.05 mg/l Indole-3-butyric acid (IBA) and 1.5 mg/l 6-benzylaminopurine (BA), pH 5.7±1 [32]. Preculture Medium 3 (PM3 original name KCMS) contains MS salts and ½ MS vitamins, 30 g/l sucrose, 8 g/l agar, 0.2 mg/l 2,4-D, 0.1 mg/l kinetin, 0.9 mg/l Thiamine hydrochloride and 200 mg/l potassium acid phosphate [33]. After two days on Preculture Media, explants were transferred to a regeneration media for induction of shoot organogenesis. Two regeneration media were used, SI-2 containing MS salts, B5 vitamins, 2 mg/l zeatin riboside trans isomer, 0.1 mg/l indole-3-acetic acid (IAA) 30 g/l sucrose, 8 g/l agar pH 5.7±1 [23]. SI-0 shoot medium containing MS salts, Nitsch & Nitsch vitamins, 2 mg/l zeatin riboside trans-isomer, 0.1 mg/l IAA 30 g/l sucrose, 8 g/l agar pH 5.7±1. Shoots were cut and transferred to a shoot elongation media containing the same components as SI-2 medium but with 1 mg/l Zeatin riboside. The elongated shoots were excised and transferred to an MS medium supplemented with 1mg/l IAA for rooting. On average 10 explants per plate were cultured, 3 plates for each treatments were used and the experiment was repeat three times. The efficiency of shoot regeneration was determined as the number of explants with shoots versus total explants. In addition, the mean of the shoots number per explants was counted.

For Agrobacterium-mediated transformation, cotyledons explants of 7 days to 10 days old tomato seedlings were incubated for 20 min in an *Agrobacterium tumefaciens* bacteria suspension (overnight culture was diluted 40 X on MS liquid medium containing 200 μM acetosyringone). Cotyledon explants were placed adaxial side down on a callus induction medium (PM).

Two days after co-cultivation on PM, cotyledons were transferred to a SI-2-Mannose shoot induction and selection medium containing MS salts, B5 vitamins, 4 g/l sucrose, 8 g/l mannose, 8 g/l plant agar, zeatin riboside, trans isomer 2 mg/l, IAA 0.1 mg/l pH 5.7±1 and 800 mg/l ticarcillin disodium/clavulanate potassium 15:1 (to eliminate *Agrobacterium*). The same SI-2 medium [23] without mannose and with 30 g/l sucrose was used as a control for tomato cotyledons organogenesis.

The first shoots appear after 4 to 6 weeks on selection medium, or after 3 weeks on SI-2 medium without mannose. Shoots were cut and transferred to a shoot elongation medium SE with or without mannose. This medium is similar to SI-2 medium but the 2 mg/l Zeatin riboside, trans-isomer was reduced to 0.5 mg/l. After 3 weeks of growth, shoots of approximately 1 to 2 cm were cut and transferred into a root induction medium containing MS salts, Nitsch & Nitsch vitamins, 30 g/l sucrose, 8 g/l agar, IAA 1 mg/l, pH 5.7±1.

### PCR analysis of transgenic plants

First, genomic DNA was isolated using the DNeasy Plant Mini Kit (Qiagen). The primers for the detection of a 1,176 bp fragment of the PMI gene were 5′-GCACTCGAGCATGCAAAAACTCATTAACTCAG-3′ and 5′-GCACTCGAGCTCTTACAGCTTGT TGTAAAC-3′. The primers for a 386 bp fragment of the Rs-AFP1-SP1-1 were: 5′-CAGGCTTCACAATGGCTAAGT-3′ and 5′-CGCGCTATATTTTGTTTTCTATC-3′. PCR amplifications from genomic DNA were done using the PCR super mix (Invitrogen) following the manufacturer´s instructions. In other cases the Extract-N-Amp Plant PCR Kits (Sigma-Aldrich) was used as described according to the manufacturer´s instructions. Amplifications were performed in a thermocycler (Eppendorf). PCR products were analyzed by electrophoresis in 1 or 2% agarose gels.

### Determination of transgene copy number by qPCR

The copy number of the transgene in different transgenic plants was determined by qPCR. The method of Salazar-Gonzales et al. [34], with some modifications, was performed. In this work, the Lat52 gene was used as an endogenous reference marker with the previously reported set of primers Lat1: 5′-AGACCACGAGAACGATATTTGC-3′ and Lat2: 5′-TTCTTGCCTTTTCATATCCAGACA-3′ [35]. qPMI-Fw: 5′-GCACGTTATCGGAGTTTGCC-3′ and qPMI-Rv: 5′-GAAGCGATGTTCCTGTTCGC-3′ primers were designed for the selection marker Phosphomannose isomerase gene (PMI) using the Primer3 tool (http://www.ncbi.nlm.nih.gov/tools/primer-blast/). The quantitative PCR was perfomed in a 7500 real-time PCR system (Applied Biosystems, Carlsbad, CA, USA). Individual PCR reaction mixtures contained 4 μl of diluted cDNA (1: 5), 10 μl of Sybr Green Mastermix (Thermo Fisher Scientific, Rockford, IL, USA), and 250 nM of each primer in a final volume of 20 μl. In all experiments, three technical replicates with same 12,5 ng of DNA were performed. The following cycling conditions were used: 95°C for 10 min (initial denaturation), 40 cycles at 95°C for 15 s for denaturation, and 60°C for 60 s for annealing and extension. Target PMI gene or endogenous Lat gene were amplified by triplicate from a T0 transgenic line T583. To determine copy number, serial dilutions of DNA (T0 of T583 line) ranging from 1.5 to 100 ng/μl were assayed in qPCR reactions. Standard curves were performed plotting the common logarithm of dilution series of DNA (100, 50, 25, 6,25, 3,124 and 1,562 ng) against the cycle threshold (Ct) values. To estimate the copy number of transgene, qRT-PCR for PMI and Lat52 genes with 12 ng of DNA from WT and from transgenic lines (T-583-4, T-583-5 and T-583-6) were performed in triplicate. The obtained Ct values were used in the following equation to calculate the transgene copy number:

$$\frac{X_{0}}{R_{0}}=10^{\left[(Ct,X-IX)÷SX]-[(Ct,R-IR)÷SR\right]}$$

where R0 is the initial amount of reference copies, X0 is the initial amount of copies of target gene, IX and IR are the intercepts SX and SR are the slopes of the relative standard curves of target and reference genes, respectively, and Ct,X and Ct,R are the Ct values of target and reference genes. The copy number of the target gene (X0) can be deduced from the Ct,X, Ct,R, SX, SR, IX, and IR values calculated from the standard curve if the copy number of the reference gene (R0) is known.

### RT-PCR analysis

Total RNA from leaves was extracted with DNeasy Plant Mini Kit (Qiagen) or mirVana® miRNA isolation kit/Ambion® Plant RNA isolation aid according to the supplier’s instructions. One microgram of RNA was reverse transcribed with the TaqMan® reverse transcription reagents (Life Technologies) with random hexamers in a 100 μl reaction. Amplification of cDNA was carried out using the RsAFP1 forward primer (5´- CAGGCTTCACAATGGCTAAGT-3´) and the attB2 reverse primer (5′-GGGGACCACTTTGTACAAGAAAGCTGGGT-3′). PCR amplifications from cDNA were done using the PCR super mix (Invitrogen) following the manufacturer´s instructions. Micro Tom actin primers were included as a quality control: Micro tom-Actin Fw: 5′-GATGGATCCTCCAATCCAGACACTGTA-3′, Micro tom-Actin-Rw: 5′-GTATTGTGTTGGACTCTGGTGATGGTGT-3′. Amplified products were analyzed via electrophoresis in 2% agarose gels stained with ethidium bromide.

### Quantitative RT-PCR

Total RNA from tomato fruits was extracted with mirVana® miRNA isolation kit and/or with Ambion® Plant RNA isolation kit according to the supplier’s instructions. One microgram of RNA was reverse transcribed with the Quantitect Reverse Transcription kit (Qiagen) following the manufacturer’s instructions. The following set of primers was used to amplify SP1-1 gene: qRsAFP-SP1-1Fw-1: 5′-AAGCTGGGTTCTACAACAATCTCT-3′, qRsAFP-SP1-1Rw-1: 5′-AAAAGCAGGCTTCACAATGGC-3′. The pathogenesis-related PR1a (P4) gene from tomato was amplified with the following set of primers: PR-Fw: 5′-CACACGACATGCAATCTCCT-3′, PR-Rw: 5′-AGTGAACACTTTTGGTTTCGAT-3′. Primer pairs were checked for amplification specificity using a serial cDNA dilution. qRT-PCR was carried out in a 7500 real-time PCR system (Applied Biosystems, Carlsbad, CA, USA) and the qPCR reaction was the same as described above. Individual PCR reaction mixtures contained 4μl of diluted cDNA (1:5), 10 μl of Sybr Green Mastermix (Thermo Fisher Scientific, Rockford, IL, USA), and 250 nM of each primer in a final volume of 20 μl. In all infection experiments, a minimum of two biological replicates for each sample and three technical replicates were performed. Normalization of real-time qRT-PCR data was performed by geometric average of two internal control genes (Le-ACTINforward: 5′-CGGTGACCACTTTCCGATCT-3′; reverse 5′-TCCTCACCGTCAGCCATTTT-3′; Le-UBIQUITIN forward: 5′-TCGTAAGGAGTGCCCTAATGCTGA-3′; reverse: 5′-CAATCGCCTCCAGCCTTGTTGTAA-3′) [36]. Significant differences relative to Mock treatment (MgCl_2_) were calculated by *t*-test analysis.

### Extraction of apoplastic fluid and determination of antibacterial activity of synthetic peptides in presence of apoplastic fluid

Apoplastic fluid was extracted using vacuum infiltration. Tomato leaves were cut from 6–8 weeks old plants, washed with distilled water and then dried using tissue paper. Five to seven leaves were placed in a 50 ml receptacle containing 30 ml of sterile deionized water. Cycles of 4 min vacuum and 10 min pause before slow release were applied in a desiccator connected to a vacuum pump until the leaves were completely infiltrated (greener leaves). The infiltrated leaves were dried with tissue towels, rolled and put into a 5 ml syringe (without cannula). The syringe was placed in a 15-ml conical falcon tube. The apoplast extract was collected by spinning the conical tubes at 2,225 g for 20 min at 4°C. Protein concentration of the apoplastic fluid was determined using the Bradford assay.

Approximately 10^6^ cfu/ml bacteria (*Xanthomonas campestris pv vesicatoria*, OD_600_ nm) were incubated with 0 or 10 mg/ml peptide in the presence or absence of 10 mg/ml of tomato apoplastic fluid. Bacterial in-vitro assays were performed in sterile flat-bottom 96-well polypropylene-plates (Greiner bio-one, Frickenhausen, Germany). After incubation at 27°C for 12–16 hours bacterial growth was analyzed by measuring the OD_600_ nm using a Tecan Genios microplate reader (Crailsheim, Germany). Experiments were done in at least three biological replicates. Differences between the mean values were analysed using an unpaired t-test analysis of variance.

### Inoculation of tomato fruits

The protocol from Zeitler et al 2012 was used in a modified form [19]. Immature unripe fruits were detached with cotton wool soaked in 80% ethanol, cut into two pieces and placed on MS-agar plates. Bacteria cells from an overnight culture were first resuspended in 10 mM MgCl_2_. After washing with 10mM MgCl_2_, the concentration of bacteria was adjusted to 0.002 OD_600_ nm (approximately 1 x 10^6^ CFU/ml) and inoculated immediately by injecting 50 μl bacteria (per spot) into tomato fruits. The fruits were injected up to ten times in different positions in transgenic or wild type tomato fruits. The fruits were kept in a humid chamber and incubated at 28°C. The infection process was monitored every day and infection sides were counted after 2–3 days. Three independent experiments from each transgenic line were performed with 4 to 6 fruits per experiment. The differences between the mean values were analyzed using an unpaired t-test analysis of variance.

## Results

### Antimicrobial activity of SP1-1 against *X*. *campestris* pv. *vesicatoria*

The rational design of antimicrobial peptides allows the optimization of the selectivity and stability which results in higher specific antimicrobial activity and reduced cytotoxic and phytotoxic effects. Microdilution assays allow a rapid determination of the antimicrobial activity of synthetic antimicrobial peptides against several different plant pathogens. These assays have been used in the past to characterize more than 60 synthetic peptides for their plant protective potential against several pathogens [19]. This study showed that the synthetic peptide SP1-1 has a low phytotoxicity, low toxicity against human blood cells and a broad range of antimicrobial activity against several plant pathogens, including *X*. *campestris* pv *vesicatoria* [19].

**Fig 1:**
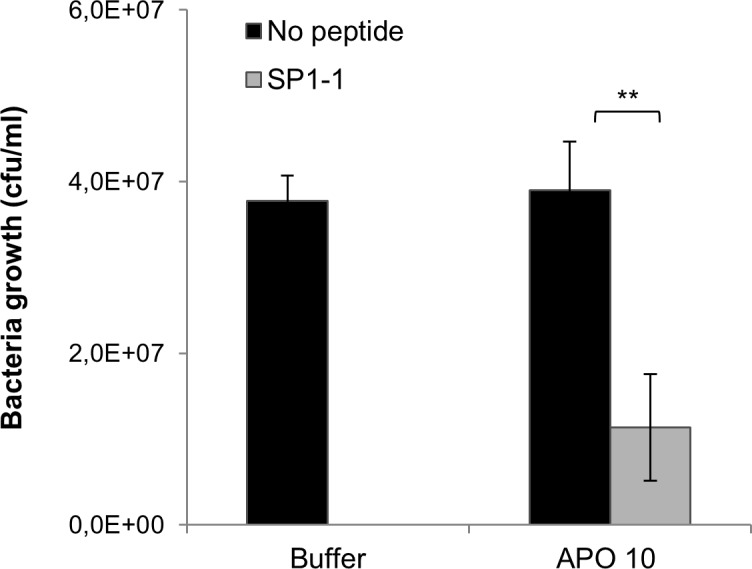
Antibacterial activity of synthetic peptide SP1-1 in the presence of tomato apoplastic fluid. *X*. *campestris* pv *vesicatoria* (10^5^ cfu/ml) was incubated with 0 or 10 μg/ml of peptide SP1-1 in the presence or absence of 10 μg/ml of tomato apoplastic fluid. Bacterial growth was determined by measuring OD_600nm_ (OD 0.2 = 10^8^ cfu/ml) 15 hours after APO; tomato apoplastic fluid. Values represent the mean of at least three biological replicates ± standard error of the mean. Asterisks indicate significant different in comparison to the corresponding control treatment, *P<0.05, **P<0.01.

*X*. *campestris* pv *vesicatoria* is a pathogen which mainly propagates in the apoplastic fluid. The presence of proteases on the surface of leaves and in the apoplastic fluid can be a limitation for the use of antimicrobial peptides for plant protection. Therefore, we first analysed the antimicrobial activity of SP1-1 in the presence of tomato apoplastic fluid (10 μg/ml) extracted from tomato leaves. 10 μg/ml of SP1-1 completely inhibited bacterial growth in the absence of apoplastic fluid. In the presence of 10 μg/ml of apoplastic fluid peptide SP1-1 reduced bacterial growth by 60% (Fig 1) demonstrating that peptide SP1-1 is only partly inactivated by apoplastic fluid and is suitable for “expression” in the apoplast.

### 4XW2 *cis*-element from parsley is induced by *X*. *campestris* pv. *vesicatoria* and Pep25 in tomato protoplasts

Constitutive expression of AMPs can be toxic for plants and can affect plant growth and physiology, as well as fertility and seed production [20]. To avoid such negative effects, we chose a synthetic 4XW2/4XS inducible plant promoter, which was cloned upstream to the cauliflower mosaic virus (CaMV) 35S minimal promoter [27]. The W2 *cis-*acting element was selected from the parsley pathogen-related gene 1 (PR1) [37], the S elements were selected from the parsley EL17 gene [28]. It has been shown that this pathogen-inducible plant promoter can be activated by several plant pathogens as well as by the synthetic peptide Pep25, which is an oligopeptide fragment of a *Phytophthora sojae* 42-kDa cell wall protein (DVTAGAEVWNQPVRGFKVYEQTEMT) [37].

**Fig 2:**
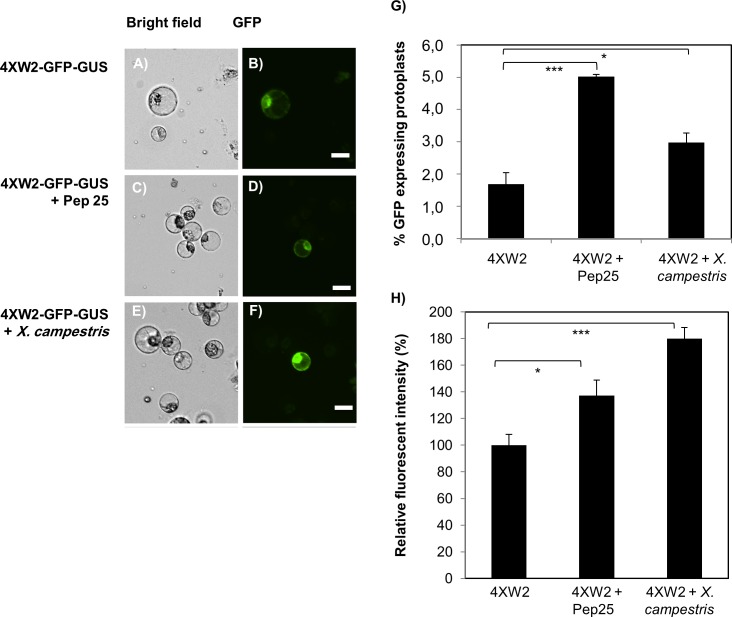
Induction of pathogen-inducible promoter 4XW2 in response to Pep25 and *X*. *campestris* pv. *vesicatoria*. The 4XW2-GFP-GUS was transiently transformed into TH3 tomato protoplasts using a modified PEG method. GFP fluorescence was monitored 12–16 h after water (A and B), pep25 (C and D) and *X*. *campestris* pv. *vesicatoria* treatment (E and F). (G) Number of protoplasts showing GFP fluorescence is given in percent. (H) Quantification of GFP fluorescence intensity was done using the Image J software (http://imagej.nih.gov/ij/). Scale bar: 20 μm. Asterisks indicate significant different in comparison to the corresponding control treatment, *P<0.05, **P<0.01, ***P<001.

To test whether the binding sites for the WRKY transcription factors (4XW2 *cis-*elements) of parsley *PR1* are functional in tomato, the promoter was first tested for expression of GFP, which was fused C-terminal to the apoplast signal peptide of the radish RsAFP1 defensine. Expression was analyzed in a transient protoplast expression system using a tomato TH3 cell line (Fig 2A–2F). The number of GFP expressing protoplasts was significantly higher after induction with Pep25 (P<0.001) and *X*. *campestris* pv. v*esicatoria* (P<0.05) (Fig 2G) and quantification of GFP fluorescence further confirmed that the 4XW2 *cis-*elements could be induced by Pep25 and *X*. *campestris* pv. *vesicatoria* (Fig 2H). However, a background activity of the promoter could be also detected in non-induced cells (Fig 2A, 2B and 2H). As a positive control for detection of GFP fluorescence, GFP was constitutively expressed in protoplast under the control of a CaMV 35S promoter (S1A and S1B Fig) [38]. No GFP signal could be detected in non-transfected cells (S1C and S1D Fig). All these experiments confirmed the functionality of the 4XW2 *cis*-elements in tomato and its inducibility by Pep25 and *X*. *campestris* pv. v*esicatoria*.

### Regeneration, transformation and characterization of Micro Tom tomato plants

For improvement of a regeneration protocol, cotyledon explants of 7 to 10 days old tomato plants (Micro Tom) were used. Pre-cultivation of cotyledons on callus induction medium (PM) for 2 to 3 days followed by 2 weeks on shoot regeneration medium significantly improved shoot regeneration (S2A Fig). For optimizing the regeneration efficiency, three pre-culture media (PM1, 2 and 3) and two shoot induction media (SI-0 and SI-2) were compared. The highest regeneration rate of up to 95% and 2–4 shoots per explants was obtained with a combination of BA and NAA in PM [39] and zeatin riboside trans-isomer and IAA in SI-2 medium (S2B–S2D Fig).

**Fig 3:**
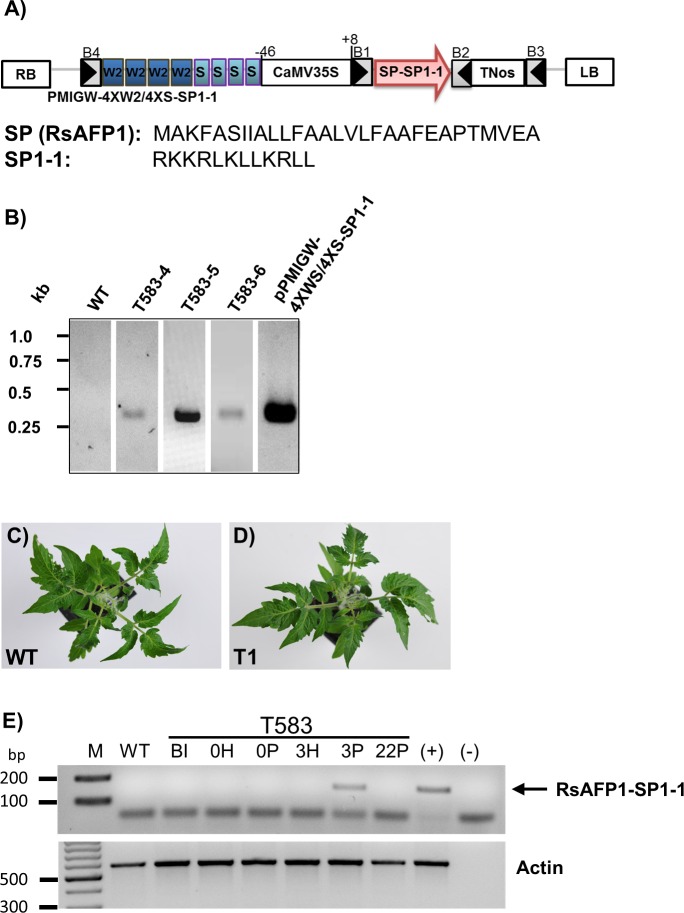
Transgenic tomato Micro Tom lines expressing the AMP SP1-1. (A) Schematic illustration of PMIGW-4XW2/4XS::SP1-1 vector construct. The vector contains a CaMV35S promoter-driven phosphomannose isomerase (PMI) gene for mannose selection. B1, B2, B3 and B4 represent attB Gateway recombination sites. SP, signal peptide RsAFP1 from radish; SP1-1, synthetic antimicrobial peptide; TNos, nopaline synthase gene terminator; RB, right border; LB, left border; W2 *cis*-acting elements from the parsley PR1 gene; S, *cis*-elements from the parsley EL17 gene; CaMV35S, minimal promoter containing the sequence -46 to +8 from the cauliflower mosaic virus 35S promoter; T35S, terminator from the cauliflower mosaic virus 35S. (B) Molecular characterization of transformed plants. PCR was done using DNA from young leaves as template and RsAFP1-TNos-specific primers. PCR fragments were obtained for T583-4, T583-5 and T583-6. PMIGW-4XW2/4XS::SP1-1 transformation vectors and WT Micro Tom tomato plants were used as positive and negative controls respectively. (C and D) Morphological phenotypes of six weeks old WT and transgenic T1 Micro Tom tomato plants. Transgenic and WT plants were grown in climate chambers for 6 weeks. (E) Detached leaves of line T583 were infiltrated with water (H) or pep25 peptide (P). Total RNA was extracted before induction (BI) and 0, 3, and 22 h after induction. RT-PCR was carried out with gene-specific primers for RsAFP1-SP1-1 and actin and fragments of 163 bp and 586 bp were expected, respectively. Genomic DNA of the transgenic line T583 was used as control (+). Total RNA from WT plants and water (-) was used as negative control. The SP1-1 band is marked with an arrow. M, 100 bp DNA ladder.

Additional exposure of regenerated shoots to IBA on a root induction medium increased root formation and the regenerated plants developed into fertile plants as shown in S2E Fig. After establishing a fast and reproducible regeneration method for Micro Tom tomato plants, the construct containing 4XW2/4XS::SP1-1 elements (Fig 3A) was introduced into cotyledons of Micro Tom plants using a *Agrobacterium-*mediated transformation approach. To avoid the uses of antibiotic or herbicide resistance marker genes for generation of tomato transgenic plants, mannose selection strategy has been chosen. The tomato plants derived from different calli were considered as independent events. In total, 14 plants were analysed. PCR amplification using primers annealing at the RsAFP1-TNos region confirmed the presence of the transgene in the lines T-583-4, T-583-5 and T-583-6, whereas no amplification was obtained in WT tomato samples (Fig 3B). To demonstrate that the signals were not from Agrobacteria the plants were analysed using primers targeting the binary vector backbone outside the T-DNA region (S3 Fig). No fragment could be amplified using extracted DNA from T583-4, T583-5 and T583-6 as the template for PCR. The copy number of the transgene in these lines was determined by qPCR as described previously [34]. In this method, standard curves were generated using serial dilutions of DNA (line T583) for an endogenous single copy gene (Lat52) and the PMI transgene (S4 Fig). To determine copy number of the transgene, DNA from each transgenic line (T583-4 to T583-6) was tested in triplicate by qPCR and the copy numbers were calculated using the corresponding standard curve. Table 1 summarizes the cycle threshold (Ct) values and the estimated copy numbers for WT and for each transgenic line. Line T583-5 has two copies of the PMI transgene, whereas the lines T583-4 and T583-6 both have a single insertion.

**Table 1:** qPCR analyses to determine the copy number of the transgene.

Transgenic line	Ct value of PMI	Ct value of Lat52	Copy number of PMI
Non-transformed	31,710 ± 0,429	21,240 ± 0,053	0,05
T583-4	29,330 ± 0,295	21,757 ± 0,117	0,98
T583-5	27,303 ± 1,508	20,933 ± 1,083	2,20
T583-6	27,543 ± 0,359	19,873 ± 0,446	0,902

Ct: cycle threshold; PMI: Phosphomannose isomerase.

**Fig 4:**
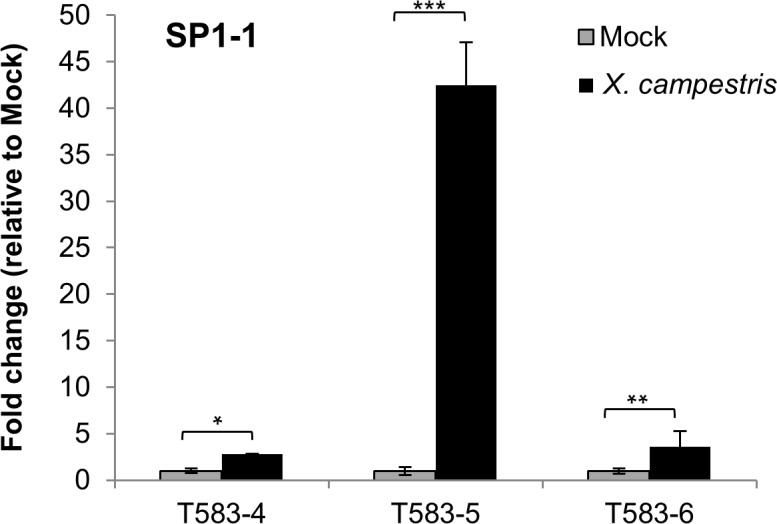
*X*. *campestris* pv. *vesicatoria* induces expression of *SP1-1* in tomato fruits. Expression of *SP1-1* was analyzed by quantitative RT-PCR in tomato fruits of T583-4, T583-5 and T583-6 36 h after inoculation and normalized to two internal reference genes (ubiquitin and actin). Expression of *SP1-1* after Mock treatment (MgCl_2_) was set to 1. Fold change of expression of *SP1-1* in *X*. *campestris* pv. *vesicatoria* treated samples is given relative to the expression in Mock treated samples. Data are the mean ±SD of two to three biological replicates. Significant differences from the control are indicated: ***, P<0.001 **, P<0.01 and *, P<0.05

Moreover, we were able to detect *SP1-1* expression in a few T1 plants, when analysing progeny of T583-5. However, the inheritance of the transgene was very low (2 of 28 analysed T1 plants), so for all further analyses T0 plants were used. Importantly, no significant difference in plant morphology, root and shoot development was observed between transgenic and wild type plants (Fig 3C and 3D). To determine the kinetic of the inducible expression of *SP1-1*, leaves from T0 transgenic plant (T583) were treated with Pep25 and were harvested at different time points (0, 3 and 22 hours). *SP1-1* expression was monitored by RT-PCR. *SP1-1* transcripts were detected in transgenic lines 3 hours after induction with Pep25 (Fig 3E). *SP1-1* expression was absent in untransformed tomato plants (negative control) and also in T583 line 22 h after induction. Additionally, we tested the induction of SP1-1 by *X*. *campestris* pv. *vesicatoria* in transgenic plants harboring PMIGW-4XW2/4XS::SP1-1. *SP1-1* transcript levels were analysed by quantitative RT-PCR in tomato fruits of T0 lines (T583-4, T583-5 and T583-6). 36 h after inoculation enhanced expression of *SP1-1* was observed in the different transgenic lines (3 to 40-fold) (Fig 4). Those results together with the Pep25 induction experiment demonstrate the functionality of transgenic lines overexpressing the SP1-1 peptide.

### Expression of SP1-1 in tomato fruits results in resistance to *X*. *campestris pv*. *vesicatoria*

**Fig 5:**
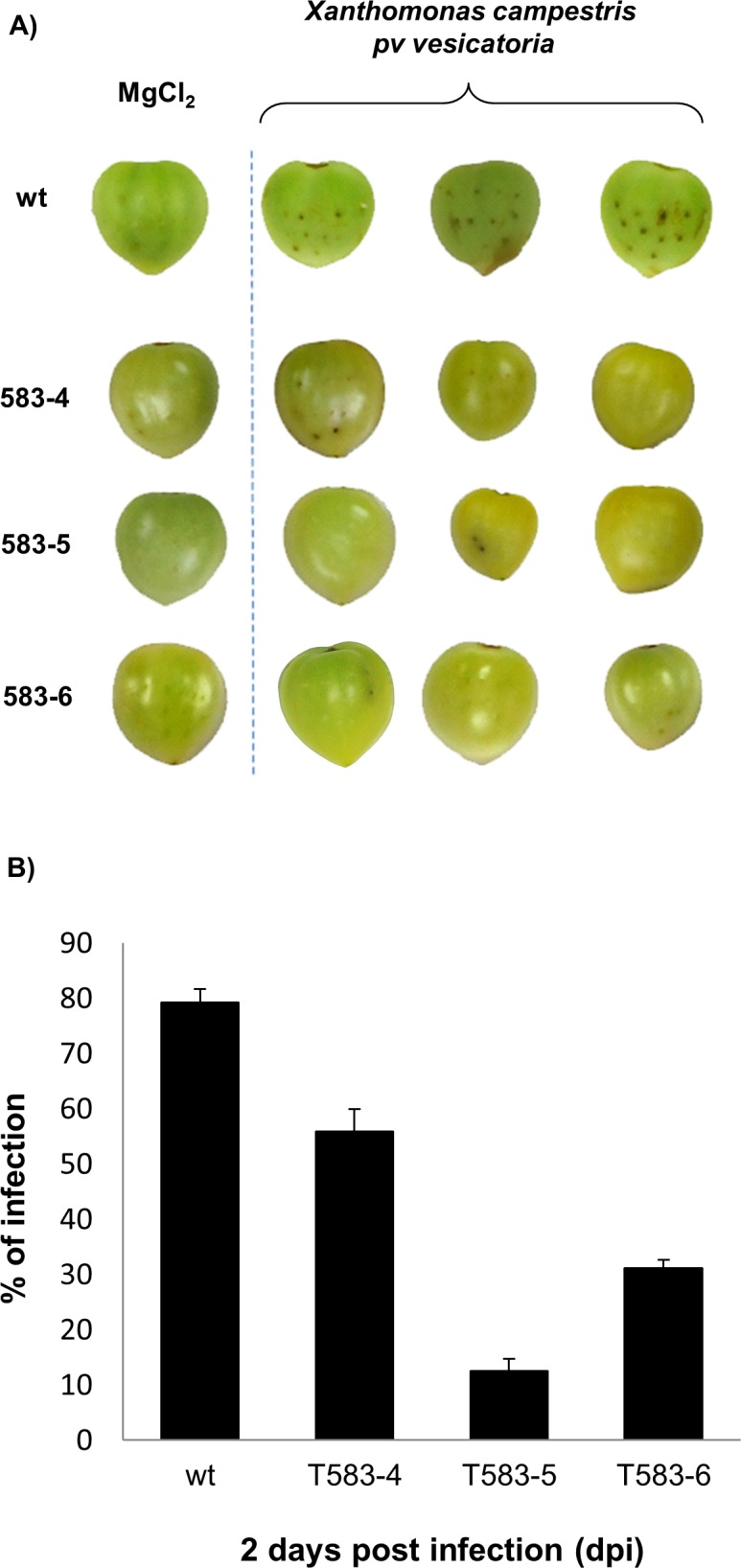
Resistance of tomato fruits of T0 transgenic plants against *X*. *campestris pv*. *vesicatoria* infection. (A) Infection symptoms of WT and transgenic T0 tomato fruits overexpressing *SP1-1* (T583-4, T583-5 and T583-6) after Mock treatment (MgCl_2_) or inoculation with *X*. *campestris* pv. *vesicatoria* (10^6^ CFU/ml). Pictures are taken two days after treatment. (B) Incidence of infection symptoms two days after inoculation with *X*. *campestris* pv. *vesicatoria* is given in percentage. The values represent the mean of three independent experiments +/- SE.

The detection of recombinant peptide SP1-1 is not possible due to lack of anti-SP1-1 antibodies for immunoblot analyses. Therefore, synthesis and accumulation of SP1-1 was confirmed by resistance analysis of the transgenic plants. Tomato fruits from WT and transgenic tomato plants were inoculated with *X*. *campestris* pv *vesicatoria* and infection symptoms of tomatoes were analyzed. For the resistance assays, plants from at least three independent transgenic lines (T583-4, T583-5 and T583-6) were selected and fruits were inoculated with *X*. *campestris* pv *vesicatoria* (Fig 5). Fruits were considered infected when browning of tissue and spot lesion formation appeared (Fig 5A). Brownish spots could be predominantly detected in fruits of WT plants two days after inoculation (infection rate of 80%). No infection was observed in mock treated tomatoes, and only a few spots were detected in transgenic lines (Fig 5A). The number of infected spots of three independent assays was quantified and infection rate is shown as the ratio of number of infected spots to total number of inoculation sites on at least three tomato fruits per experiment (Fig 5B). For lines T583-4, T583-5 and T583-6 infection rates of 55,83%, 12,50% and 31,11%, respectively, were determined. Disease development over 3 days and disease scoring for WT fruits and fruits of two T0 lines (T583-4 and T583-5) is shown in S5 Fig. To demonstrate that the transgenic plants show minimal immune response upon bacterial challenge, expression of pathogenesis related gene 1 (PR1) was analysed in the three different transgenic lines (S6 Fig). Slightly enhanced expression of *PR1* could be observed in all three transgenic lines after bacteria challenge (1,5 to 2,5 fold change induction). However, a significant difference in *PR1* expression between treated and untreated fruits (P<0,01) could be only observed in T583-5 plants, concluding that in general plant immune response upon bacterial challenge is quite low. Taken together, these results indicate that expression of SP1-1 significantly improves resistance in tomato fruit resistance against *Xanthomonas vesicatoria* pv *campestris* infection.

## Discussion

Plant diseases, alongside climate change and soil erosion, are the leading cause of diminished crop yields worldwide. On average, at least 10% of crop yields are lost around the world due to plant infections [40]. Chemical control of plant pathogens is widely used, as modern synthetic chemicals are less toxic than traditional copper based pesticides. However, modern pesticides are expensive and several plant pathogens can adapt and develop resistance to them [41,42]. A promising approach to combat plant pathogens is the use of antimicrobial peptides (AMPs) in engineered plants [43–45]. AMPs can be optimized to be more stable and more specific against distinct pathogens [46].

In this study, transgenic tomato plants expressing the *de-novo* designed AMP SP1-1 under control of a pathogen inducible promoter were generated. We choose SP1-1 because this peptide has been optimized in terms of activity, specificity and reduced toxicity against human blood and plant cells [19]. In addition, a protocol for plant based production and purification of a biologically active derivative of SP1-1 (SP-1) produced in *Nicotiana benthamiana* plants has been described [47]. In general, AMPs can be optimized to display high antibacterial activity [16,48]. Thus, SP1-1 as a derivative of SP1 was identified by an optimization screen with 22 variations of SP1. Importantly, SP1-1 shows the highest antimicrobial activity among all SP1 derivatives. A single amino acid exchange from valine to leucine at the C-terminus of SP1 resulted in a more than 20 times higher growth inhibition of *Xanthomonas campestris* pv. *vesicatoria*.

Another important property of AMPs, which has to be considered for generation of transgenic plants, is the stability against degradation through endogenous host proteases. Previous studies with transgenic tobacco plants expressing a ceropin-based peptide showed almost no resistance against *Pseudomonas solanacearum* and *Pseudomonas syringae* pv *tabaci* although the plants were expressing the AMP. This surprising result could be explained by the degradation of the peptide by endogenous host proteases [49,50]. We demonstrated the stability of SP1-1 against degradation by *Xanthomonas campestris* pv. *vesicatoria* or tomato plant-apoplast fluid (Fig 1).

Inducible expression of AMPs is a promising strategy, especially in the case, that the produced AMPs display a phytotoxic activity. Using this strategy the phytotoxic AMP BP100 was produced in rice under the control of a heat shock inducible promoter. The generated rice plants showed moderate yields of BP100 after exposure to high temperatures and NaCl stress [22]. Furthermore, it could be demonstrated that the potential negative effects of produced phytotoxic AMPs is limited to the duration and sites of expression. In this way, negative effects on plant development and potential exposure of AMPs to non-pathological bacteria strains is reduced [22]. Another example of pathogen-inducible expression of AMPs in plants is presented in from studies in tobacco using a *win3*.*12T* pathogen-inducible promoter from poplar [21, 51]. In these studies CEMA, a cecropin A-melittin hybrid peptide, and two AMPs derived from frog skin, MsrA2 and temporin A, have been expressed in tobacco plants. Transgenic tobacco plants were resistant against several phytopathogenic fungi, such as *Fusarium solani*, *F*. *oxysporum*, *Alternaria alternata*, *Botrytis cinerea* and *Sclerotinia sclerotiorum*. Additionally, no toxic effects on plant growth and development could be observed in these transgenic plants [21,51]. Interestingly, the pathogen-inducible promoter *win3*.*12T* also contained W *cis*-elements. Several studies have demonstrated that W *cis*-elements are present in different plant species and that their nucleotide sequences are highly conserved among plants [52–54]. Based on this, the synthetic pathogen-inducible promoter 4XW2/4XS containing tetramers of W and S *cis*-elements from parsley has been chosen in this study. It has been shown that these W and S *cis*-elements can be activated by a wide range of plant pathogens in transgenic *Arabidopsis* plants and that the gene expression is only induced locally at the side of infection [27].

The functionality of the 4XW2/4XS promotor in tomato transgenic plants by pathogens could be confirmed in three different ways. Firstly, RNA transcripts could be detected in the engineered tomato plants after induction with Pep25, an oligopeptide fragment of a *Phytophthora sojae* (42-kDa cell wall protein). Secondly, the functionality of 4XW2 *cis*-elements could be demonstrated by transient expression of GFP in tomato TH3 protoplasts. Interestingly, the protoplasts showed a low background expression in non-induced cells, which can be explained by a stress response to protoplast preparation. Previous reports in *Arabidopsis* demonstrated that W2 and S *cis*-elements from the PR1 and the EL17 promoter show both a low level of background expression and an induction of transcription by wounding [27]. Thirdly, tomato fruits of transgenic plants were more resistant to infection with *X*. *campestris* pv. v*esicatoria* than WT plants (Fig 5). Regarding the morphological phenotype of the transgenic tomato plants, no difference in growth and development could be observed in comparison to WT plants (Fig 3C and 3B). Furthermore, no difference in terms of germination rate and seed viability was observed. These results confirm previous reports, that inducible expression of toxic peptides does not significantly affect plant growth and development [21,22].

In summation, we generated transgenic tomato plants expressing an AMP a under control of an inducible promoter without using an antibiotic-resistance marker. 4XW2/4XS *cis*-elements from parsley in combination with the AMP SP1-1 appears to be a promising alternative for protection of tomato plants/fruits against *Xanthomonas campestris* pv. *vesicatoria* infections. However, field experiments have to be done in the future to assess, if this strategy is also effective in the natural environment.

## Supporting Information

S1 Fig Microscopic pictures of transient transformed protoplasts. GFP construct containing the CaMV 35S promoter as a positive control (A-B) were transiently transformed into TH3 tomato protoplasts using a modified PEG method. GFP fluorescence was analysed 12 h after Pep25 treatment (A). Non-transformed protoplasts were used as a negative control (C-D). Scale bar: 25 μm.
(PDF)

S2 Fig Regeneration of tomato Micro Tom plants from cotyledons. (A) Tomato seedlings growing in MS medium. (B) Shoot formation was induced on PM/SI-2 media after 4 weeks. (C) Callus and shoot formation was induced on PM3/SI-2 media after 4 weeks. (D) Plant regeneration rate in percent. (E) Fertile regenerated plants growing in a climate chamber.
(PDF)

S3 Fig Prove that T1 lines do not contain Agrobacterium. A PCR was performed using primers targeting the binary vector backbone. The following primers have been used: Ext-Flanking-Forward 5`-GAAGCCATGAAAACCGCCAC-3`; Ext-Flanking-Reverse 5`-GCCTGTCGCGTAACTTAGGA-3`. The binary vector pMIGW7/SP1-1 was used as positive control.
(PDF)

S4 Fig **Representative standard curves of Real-time PCR amplification of the Phosphomannose isomerase (PMI) gene (A) and the Lat52 endogenous gene (B).** Representative standard curve were obtained from the amplification of twofold serial dilutions of DNA from line T583. Axis: Cycle threshold (Ct) value versus the logarithmic concentration (ng) of total DNA. Dark dots represent the 6 points analyzed by triplicate.
(PDF)

S5 Fig Disease development and disease scoring of WT and SP1-1 producing transgenic tomato fruits after inoculation with *X*. *campestris pv*. *vesicatoria*. (A) Disease development in wild-type (WT) and T1 transgenic tomato fruits carrying the transgene SP1-1 (T583-4, T583-5), after mock treatment (MgCl_2_) or 1, 2 and 3 days after inoculation with *X*. *campestris* pv. *vesicatoria*. (B) Incidence of infection symptoms 1, 2 and 3 days after inoculation with *X*. *campestris* pv. *vesicatoria* is given in percentage. The values represent the mean of three independent experiments +/- SE.
(PDF)

S6 Fig Pathogenesis-related 1 (PR1) gene expression in transgenic tomato fruits after *X*. *campestris* pv. *vesicatoria*. Expression of *PR1* was analyzed by quantitative RT-PCR in tomato fruits of T583-4, T583-5 and T583-6 36 h after inoculation with *X*. *campestris* pv. *vesicatoria* and normalized to two internal reference genes (ubiquitin and actin). Expression of *PR1* after Mock treatment (MgCl_2_) was set to 1. Fold change of expression of *PR1* in *X*. *campestris* pv. *vesicatoria* treated samples is given relative to the expression in Mock treated samples. Data are the mean ±SD of two to three biological replicates. Significant differences from the control are indicated: ***, P<0.001 **, P<0.01 and *, P<0.05.
(PDF)
