# Supplementary material for: Inducible Expression of the De-Novo Designed Antimicrobial Peptide SP1-1 in Tomato Confers Resistance to Xanthomonas campestris pv. vesicatoria
Source: PLoS One. 2016 Oct 5;11(10):e0164097. doi: 10.1371/journal.pone.0164097 (PMC5051901; doi:10.1371/journal.pone.0164097)
Supplement: S1 Fig — GFP construct containing the CaMV 35S promoter as a positive control (A-B) were transiently transformed into TH3 tomato protoplasts using a modified PEG method. GFP fluorescence was analysed 12 h after Pep25 treatment (A). Non-transformed protoplasts were used as a negative control (C-D). Scale bar: 25 μm. (PDF) [file pone.0164097.s001.pdf]

S1 Supporting Information

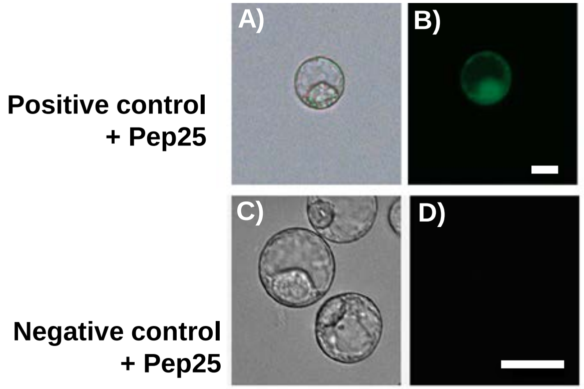

S1 Fig. Microscopic pictures of transient transformed protoplasts. GFP construct containing the CaMV 35S promoter as a positive control (A-B) were transiently transformed into TH3 tomato protoplasts using a modified PEG method. GFP fluorescence was analysed 12 h after Pep25 treatment (A). Non-transformed protoplasts were used as a negative control (C-D). Scale bar: 25 µm.
