# Supplementary material for: Inducible Expression of the De-Novo Designed Antimicrobial Peptide SP1-1 in Tomato Confers Resistance to Xanthomonas campestris pv. vesicatoria
Source: PLoS One. 2016 Oct 5;11(10):e0164097. doi: 10.1371/journal.pone.0164097 (PMC5051901; doi:10.1371/journal.pone.0164097)
Supplement: S2 Fig — (A) Tomato seedlings growing in MS medium. (B) Shoot formation was induced on PM/SI-2 media after 4 weeks. (C) Callus and shoot formation was induced on PM3/SI-2 media after 4 weeks. (D) Plant regeneration rate in percent. (E) Fertile regenerated plants growing in a climate chamber. (PDF) [file pone.0164097.s002.pdf]

S2 Supporting Information

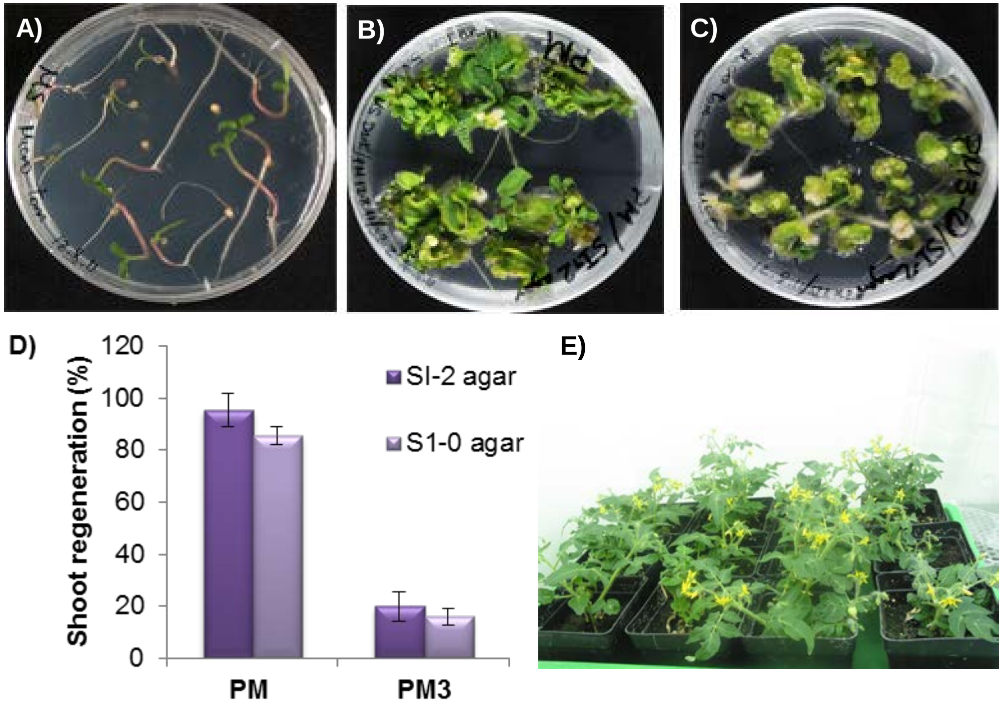

S2 Fig. Regeneration of tomato Micro Tom plants from cotyledons. (A) Tomato seedlings growing in MS medium. (B) Shoot formation was induced on PM/SI-2 media after 4 weeks. (C) Callus and shoot formation was induced on PM3/SI-2 media after 4 weeks. (D) Plant regeneration rate in percent. (E) Fertile regenerated plants growing in a climate chamber.
