# Supplementary material for: Inducible Expression of the De-Novo Designed Antimicrobial Peptide SP1-1 in Tomato Confers Resistance to Xanthomonas campestris pv. vesicatoria
Source: PLoS One. 2016 Oct 5;11(10):e0164097. doi: 10.1371/journal.pone.0164097 (PMC5051901; doi:10.1371/journal.pone.0164097)
Supplement: S3 Fig — A PCR was performed using primers targeting the binary vector backbone. The following primers have been used: Ext-Flanking-Forward 5`-GAAGCCATGAAAACCGCCAC-3`; Ext-Flanking-Reverse 5`-GCCTGTCGCGTAACTTAGGA-3`. The binary vector pMIGW7/SP1-1 was used as positive control. (PDF) [file pone.0164097.s003.pdf]

S3 Supporting Information

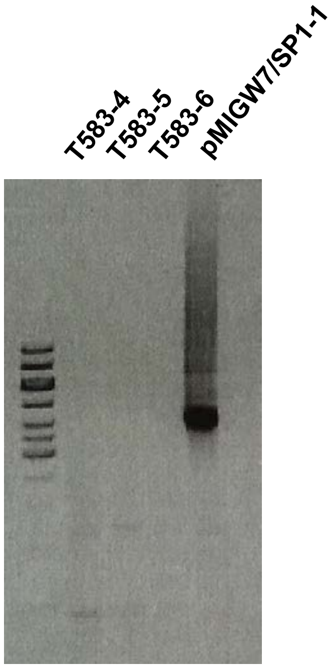

S3 Fig. Prove that T1 lines do not contain Agrobacterium. A PCR was performed using primers targeting the binary vector backbone. The following primers have been used: Ext-Flanking-Forward 5`-GAAGCCATGAAAACCGCCAC-3`; Ext-Flanking-Reverse 5`-GCCTGTCGCGTAACTTAGGA-3`. The binary vector pMIGW7/SP1-1 was used as positive control.
