# Supplementary material for: Inducible Expression of the De-Novo Designed Antimicrobial Peptide SP1-1 in Tomato Confers Resistance to Xanthomonas campestris pv. vesicatoria
Source: PLoS One. 2016 Oct 5;11(10):e0164097. doi: 10.1371/journal.pone.0164097 (PMC5051901; doi:10.1371/journal.pone.0164097)
Supplement: S4 Fig — Representative standard curves of Real-time PCR amplification of the Phosphomannose isomerase (PMI) gene (A) and the Lat52 endogenous gene (B). Representative standard curve were obtained from the amplification of twofold serial dilutions of DNA from line T583. Axis: Cycle threshold (Ct) value versus the logarithmic concentration (ng) of total DNA. Dark dots represent the 6 points analyzed by triplicate. (PDF) [file pone.0164097.s004.pdf]

# S4 Supporting Information

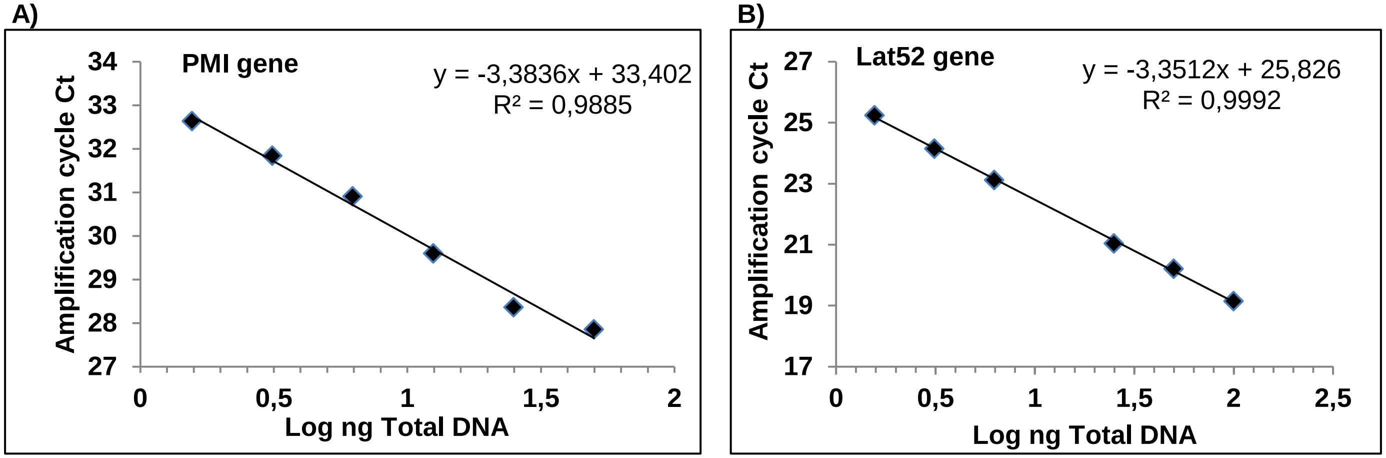


**S4 Fig. Representative standard curves of Real-time PCR amplification of the Phosphomannose isomerase (PMI) gene (A) and the Lat52 endogenous gene (B).** Representative standard curve were obtained from the amplification of twofold serial dilutions of DNA from line T583. Axis: Cycle threshold (Ct) value versus the logarithmic concentration (ng) of total DNA. Dark dots represent the 6 points analyzed by triplicate.
