# Supplementary material for: Inducible Expression of the De-Novo Designed Antimicrobial Peptide SP1-1 in Tomato Confers Resistance to Xanthomonas campestris pv. vesicatoria
Source: PLoS One. 2016 Oct 5;11(10):e0164097. doi: 10.1371/journal.pone.0164097 (PMC5051901; doi:10.1371/journal.pone.0164097)
Supplement: S5 Fig — (A) Disease development in wild-type (WT) and T1 transgenic tomato fruits carrying the transgene SP1-1 (T583-4, T583-5), after mock treatment (MgCl2) or 1, 2 and 3 days after inoculation with X. campestris pv. vesicatoria. (B) Incidence of infection symptoms 1, 2 and 3 days after inoculation with X. campestris pv. vesicatoria is given in percentage. The values represent the mean of three independent experiments +/- SE. (PDF) [file pone.0164097.s005.pdf]

S5 Supporting Information

A)

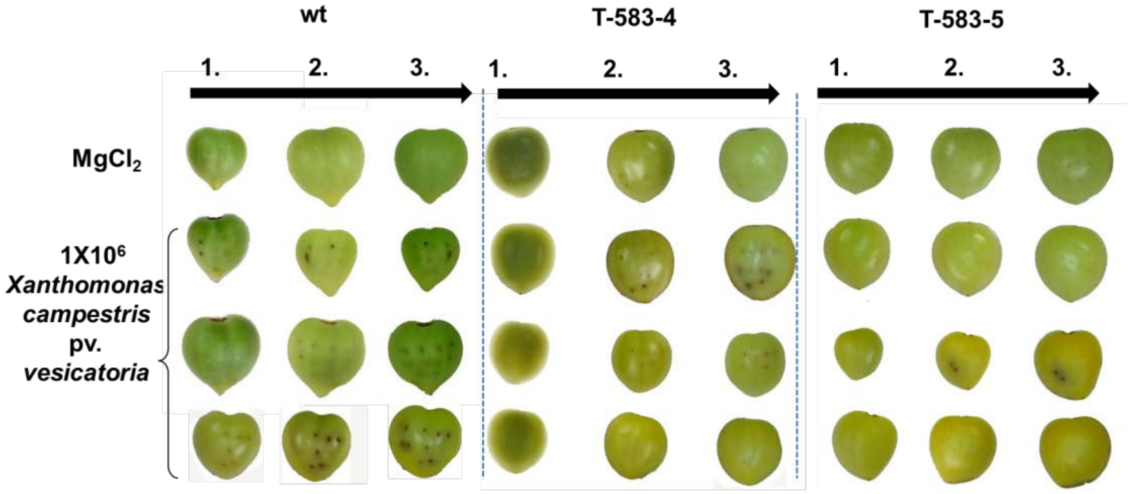

B)

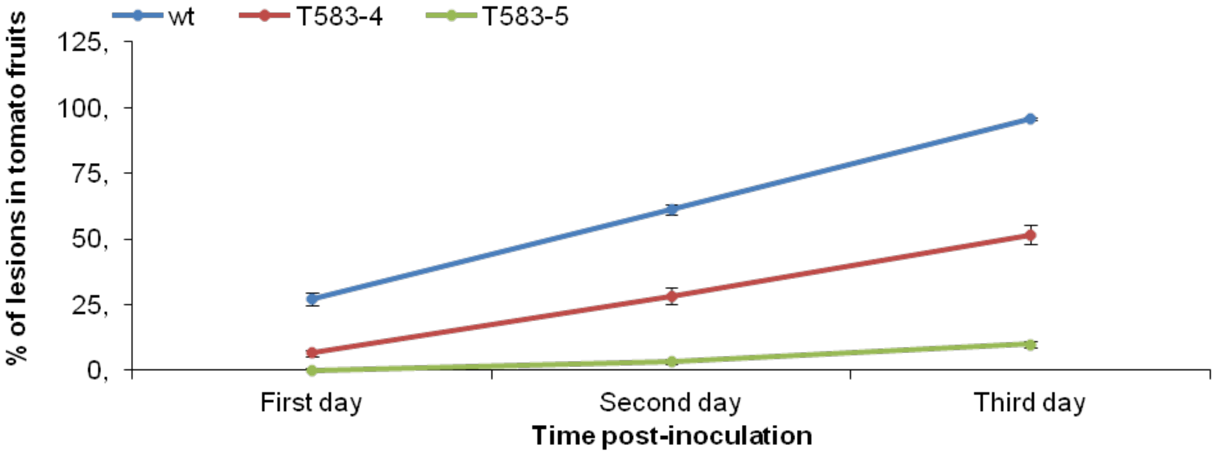

S5 Fig. Disease development and disease scoring of WT and SP1-1 producing transgenic tomato fruits after inoculation with *X. campestris pv. vesicatoria*. (A) Disease development in wild-type (WT) and T1 transgenic tomato fruits carrying the transgene SP1-1 (T583-4, T583-5), after mock treatment ($MgCl_2$) or 1, 2 and 3 days after inoculation with *X. campestris* pv. *vesicatoria*. (B) Incidence of infection symptoms 1, 2 and 3 days after inoculation with *X. campestris* pv. *vesicatoria* is given in percentage. The values represent the mean of three independent experiments +/- SE.
