# Supplementary material for: Inducible Expression of the De-Novo Designed Antimicrobial Peptide SP1-1 in Tomato Confers Resistance to Xanthomonas campestris pv. vesicatoria
Source: PLoS One. 2016 Oct 5;11(10):e0164097. doi: 10.1371/journal.pone.0164097 (PMC5051901; doi:10.1371/journal.pone.0164097)
Supplement: S6 Fig — Expression of PR1 was analyzed by quantitative RT-PCR in tomato fruits of T583-4, T583-5 and T583-6 36 h after inoculation with X. campestris pv. vesicatoria and normalized to two internal reference genes (ubiquitin and actin). Expression of PR1 after Mock treatment (MgCl2) was set to 1. Fold change of expression of PR1 in X. campestris pv. vesicatoria treated samples is given relative to the expression in Mock treated samples. Data are the mean ±SD of two to three biological replicates. Significant differences from the control are indicated: ***, P<0.001 **, P<0.01 and *, P<0.05. (PDF) [file pone.0164097.s006.pdf]

S6 Supporting Information

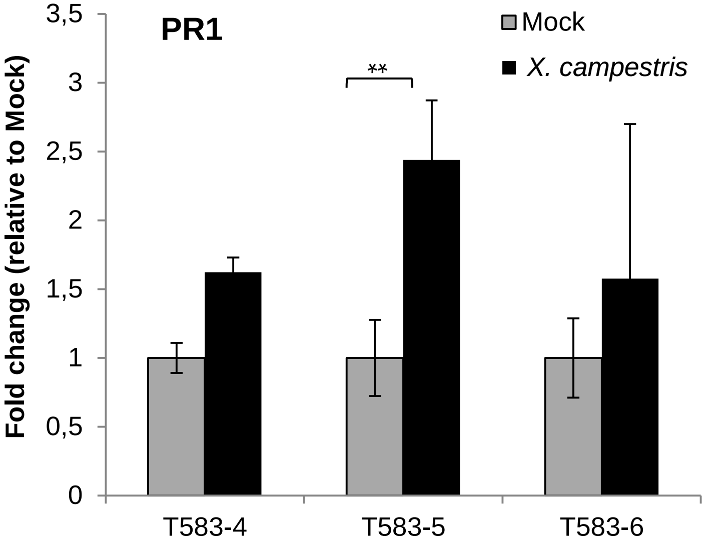

S6 Fig. Pathogenesis-related 1 (PR1) gene expression in transgenic tomato fruits after *X. campestris* pv. *vesicatoria*. Expression of *PR1* was analyzed by quantitative RT-PCR in tomato fruits of T583-4, T583-5 and T583-6 36 h after inoculation with *X. campestris* pv. *vesicatoria* and normalized to two internal reference genes (ubiquitin and actin). Expression of *PR1* after Mock treatment ($MgCl_2$) was set to 1. Fold change of expression of *PR1* in *X. campestris* pv. *vesicatoria* treated samples is given relative to the expression in Mock treated samples. Data are the mean ±SD of two to three biological replicates. Significant differences from the control are indicated: ***, P<0.001 **, P<0.01 and *, P<0.05
